# Collaborating for Impact: Navigating Partnerships and Overcoming Challenges across the Sustainable Development Goals

**DOI:** 10.1021/acssuschemeng.4c10171

**Published:** 2025-01-16

**Authors:** Hiba Azim, Amy-Louise Johnston, Morag Nixon, John Luke Woodliffe, Romano Theunissen, Reshma Suresh, Subarna Sivapalan, Jack Bobo, Peter Licence

**Affiliations:** † School of Chemistry, University of Nottingham, Nottingham NG7 2RD, United Kingdom; ‡ Faculty of Engineering, University of Nottingham, Nottingham NG7 2RD, United Kingdom; § InsightPact, Klongton, Khlong Toei, Bangkok 10110, Thailand; ∥ Amrita School for Sustainable Development, Amrita Vishwa Vidyapeetham, Amritapuri, Kollam 690525, Kerala, India; ⊥ School of Education, Faculty of Arts and Social Sciences, University of Nottingham, Jalan Broga, 43500 Semenyih, Malaysia; # Food Systems Institute, University of Nottingham, Nottingham NG7 2RD, United Kingdom

**Keywords:** Early career researcher, Global South, SDG17, Interdisciplinary collaboration, Equitable partnerships

## Abstract

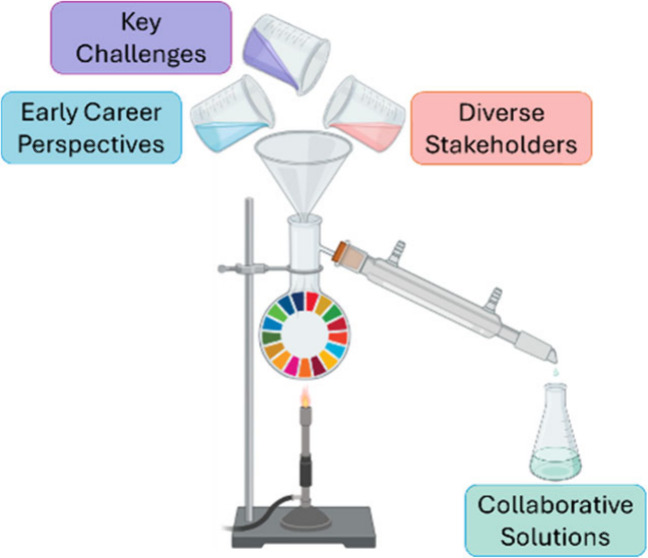

We illustrate the importance of early career perspectives and diverse partnerships to develop solutions and overcome key challenges to achieve the Sustainable Development Goals.

## 1 Introduction

The Sustainable Development Goals (SDGs), adopted by all United Nations (UN) Member States in 2015, represent a universal call to action to end poverty, protect the planet, and ensure that all people enjoy peace and prosperity by 2030. The 17 interconnected goals address global challenges, providing a shared blueprint for present and future well-being, recognizing that ending poverty must go hand-in-hand with building economic growth and addressing social needs including education, health, social protection, and job opportunities, while tackling climate change and environmental protection.^1^

At the heart of the SDGs lies SDG 17: *Partnerships for the Goals*. This goal underpins all others by recognizing that the ambitious targets set out in the 2030 agenda can only be realized with strong global partnerships and cooperation. The significance of SDG 17 cannot be overstated. It acknowledges that in our interconnected world, challenges rarely respect national boundaries, and solutions often require coordinated international efforts. This goal promotes international support for implementing effective and targeted capacity-building in developing countries, enhancing North–south, South–South, and triangular regional and international cooperation on access to science, technology, and innovation. Furthermore, it encourages the promotion of development, transfer, dissemination, and diffusion of environmentally sound technologies to developing countries on favorable terms. This paper seeks to provide a voice to the discussion from a higher education perspective exploring some of the specific challenges and opportunities that pave the road toward the realization of this goal.

The SDGs, particularly when viewed through the lens of SDG 17, are profoundly relevant to sustainability innovation within chemistry and engineering. These fields are at the forefront of developing solutions to many of the challenges addressed by the SDGs. For instance, chemists and engineers play crucial roles in developing clean energy technologies as outlined in *Affordable and Clean Energy* (SDG 7), contributing to solutions required for *Responsible Consumption and Production* (SDG 12), and innovating water purification and management systems required for *Clean Water and Sanitation* (SDG 6).

The concept of green chemistry aligns closely with the SDGs, as has been widely discussed.^2,3^ Green chemistry principles, such as designing safer chemicals and processes, using renewable feedstocks, and improving energy efficiency, directly contribute to multiple SDGs.^4,5^ Similarly, in engineering, the growing field of sustainable engineering focuses on designing and operating systems that use energy and resources at a rate that does not compromise the natural environment or the ability of future generations to meet their own needs.^6,7^

The relevance of these themes to early career researchers (ECRs) in chemistry, engineering, and related fields is profound. As the next generation of scientists and innovators, ECRs are uniquely positioned to drive forward the agenda of sustainable development.^8^ They bring fresh perspectives, new ideas, and often a strong passion for addressing global challenges. However, they also face significant challenges in this pursuit.

One major challenge for ECRs is the need to navigate the complex, interdisciplinary nature of sustainability research.^9^ The SDGs are inherently interconnected, requiring researchers to think beyond traditional disciplinary boundaries. Another challenge is the long-term holistic nature of sustainability research, which can conflict with the pressure to produce quick, publishable results often favored in academic career progression.^10^ Research has shown that ECRs may find it difficult to secure funding for projects with longer time horizons hence limiting the real-world impact of their work.^11^

Given these challenges, it is important ECRs begin collaborations early in their academic careers, providing them with a multitude of benefits. They enable the building of diverse networks that can offer support, expertise, and resources throughout their careers. These collaborations also provide invaluable exposure to different research cultures, methodologies, and perspectives, broadening the researcher’s horizons and enriching their work. This offers an opportunity to develop essential soft skills necessary for effective teamwork and project management, which are crucial in addressing complex sustainability challenges. Moreover, collaborative work often increases the chances of securing funding through joint grant applications, providing vital resources for research. ECRs should consider the global implications and applications of their research, ensuring that their work has relevance and impact beyond their immediate context. Engaging with diverse stakeholders, including those from different cultural and socioeconomic backgrounds, is crucial for developing solutions that are adaptable and effective in various settings. Perhaps most importantly, early collaborations allow ECRs to contribute to and benefit from the kind of interdisciplinary work that underpins the complex, interconnected challenges outlined in the SDGs.

This global approach naturally leads to the consideration of the relationship between the Global South and Global North. The terms “Global South” and “Global North” are used to describe broad socio-economic and political divides, roughly capturing the distinction between economically developed countries (Global North) and developing or less developed countries (Global South).^12^ It is important to note that these terms are not strictly geographical and are more about economic relationships than cardinal directions. The Global South, which includes many countries in Africa, Latin America, and parts of Asia, is disproportionately affected by many of the challenges addressed by the SDGs. The Global North has been responsible for the majority of historical greenhouse gas emissions, the impacts of climate change are often felt more severely in the Global South.^13^ Many countries in the Global South face increased vulnerability to extreme weather events, sea-level rise, and changes in agricultural productivity. Similarly, issues of poverty, lack of access to education and healthcare, and exposure to environmental pollutants are often more acute in the Global South. However, many countries in the Global South are also at the forefront of innovative solutions to sustainability challenges. For instance, some are leapfrogging outdated technologies to implement renewable energy systems, while others are pioneering nature-based solutions to climate change.^14^

For ECRs, understanding the dynamics between the Global North and South is crucial and informs several important considerations in their work. It highlights the need for equitable partnerships that recognize and value the knowledge and innovations coming from the Global South, moving beyond outdated paradigms of unidirectional knowledge transfer.

**Figure 1:**
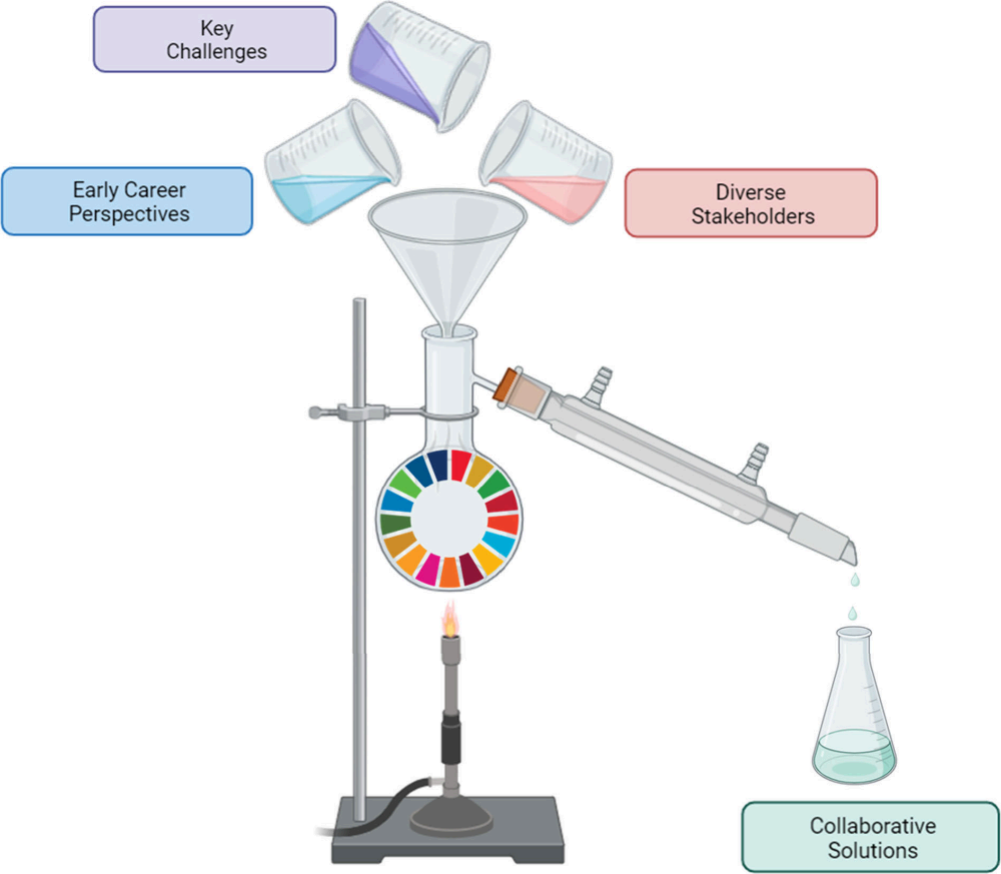
Illustrating the importance of partnerships and collaboration to develop solutions and overcome challenges to achieve the United Nations Sustainable Development Goals.

In this paper we present an example of such a perspective drawn from the experiences of four ECRs within chemistry and engineering, exploring the relevance of SDG 17 in their specific fields. Key challenges holding back potential innovation are then considered alongside possible solutions. These perspectives are then further developed in collaboration with other multidisciplinary stakeholders facilitating the distillation of four significant themes which encompass the key challenges and potential solutions which unite the diverse and global group, illustrated in Figure 1. While the conclusions drawn are broad and context dependent, we illustrate an example of how SDG 17 can be used practically toward collaborative solutions.

## 2 ECR Perspectives of Collaboration in Sustainability Research

### 2.1 Development of Advanced Wastewater Treatment Technologies

Water pollution is ambiguous in the aqueous environment, with a wide range of pollutants being observed in waters globally. Pharmaceuticals are a class of pollutants which are not removed through conventional wastewater treatment technologies, with reports of removal reaching ∼10% removal in some settings.^15^ Therefore, to facilitate removal of pharmaceuticals and other emerging pollutants, sewage treatment works need to be upgraded to include additional technologies targeting their removal and/or degradation. The presence of pharmaceuticals in the environment, especially antibiotics, is known to be having detrimental impacts on human and environmental health. Specifically, antibiotics are known to be contributing to the spread of antimicrobial resistance which is predicted to be a leading cause of death globally.^16,17^ Developing new advanced water treatment technologies for the removal of such pollutants contributes to several UN SDGs including *Good Health and Well-being* (SDG 3), *Clean Water and Sanitation* (SDG 6), and *Responsible Consumption and Production* (SDG 12).

Sorption is an advanced water treatment technology which is gaining a lot of interest as it is a technology which aligns with the requirements of a circular economy. It can also be low energy and simple if designed well. Activated carbon is the most commonly utilized sorbent material, but has limitations, especially around regeneration costs.^18^ Hence there is a drive to develop new sorbent materials such as layered double hydroxides (LDH) which are clay-like materials.^19^ It is possible to synthesize a range of LDH using a continuous flow system which has been developed at University of Nottingham (UoN).^20−23^

The multidisciplinary challenges associated with the development and evaluation of performance of new sorbent material requires a collaborative approach. A collaborative approach ensures the development of new sorbent materials produces data to further support their industrial application and technology transfer. Research areas to consider include evaluating removal of environmentally significant pollutants, such as pharmaceuticals,^19^ at environmentally relevant concentrations, typically < mg/L concentrations. Suitable analytical techniques for this work are inherently sensitive, often requiring specialist knowledge, hence collaborations are beneficial to ensure collection of robust data.^24^ Removal performance from complex environmental waters, such as municipal wastewater effluent, also provide insights into how materials will perform in industrial settings.^25^ For this, researchers require access to environmental water for sampling on a regular basis, possibly provided through relationships with water treatment facilities.

### 2.2 An Innovative Solution for Carbon Capture

Climate change is one of the greatest challenges currently facing humankind, predicted to cause significant negative impacts on our society and the environment. An essential mitigation strategy is to capture carbon dioxide (CO_2_) from its largest source: power plants. However, current amine scrubbing technologies for CO_2_ capture are ineffective due to very high energy requirements.^26^ A new class of materials known as magnetic framework composites (MFCs) have been recently explored at UoN to address the technical challenges of amine scrubbing. These novel materials achieve both a higher capacity and selectivity for CO_2_ over other gases, with much lower energies required to regenerate the sorbents for reuse using efficient magnetic induction heating.^27,28^

This research directly addresses the UN SDG of *Climate Action* (SDG 13). However, due to the impacts of climate change around the world, mitigating global warming also impacts a variety of other SDGs, such as *No Poverty* (SDG 1) and *Zero Hunger* (SDG 2).

The scale and imminence of the climate change challenge requires effective partnerships across sectors and industries. A challenge for ECRs is collaborating with industrial partners to advance academic research toward impact, particularly difficult in highly commercial fields such as CO_2_ capture.^29,30^ Fear of losing or splitting intellectual property (IP) from any joint discoveries can be enough to make industrial partners hesitate before starting collaborations with ECRs at academic institutions. The limited resources of start-up companies can also make them unable to fund contract research at universities. Understandably, in order to protect their IP, companies then require lengthy nondisclosure agreements (NDAs) and contracts to be set up between the institutions. However, the limited time scales of ECRs on short-term contracts makes it difficult to progress these collaborations, as contracts can take significant amounts of time to set up.

Helpful strategies for overcoming these barriers include IP frameworks, where academic institutions are clear and upfront about how any joint research would impact current or forward IP. Additionally, simplified or generalized NDAs which could be quickly put in place to allow collaborative research to begin would highly benefit ECRs looking to establish new collaborations with industry.

### 2.3 The Early Development of Biological Recycling of Plastic

Plastic is a versatile and valuable resource which has meant that global production continues to increase, with currently over 400 million tonnes produced annually.^31^ The durable properties of plastic has led to an increasing disposal challenge, with limited end-of-life treatment options.^32^ Plastic waste is overwhelming the waste system, resulting in waste escaping and polluting the environment, on land, in rivers and in the oceans; it is estimated 4.8 to 12.7 million tonnes entered the ocean in 2010.^33^

Plastic waste can be managed using several waste streams including recycling; mechanical, chemical or biological, energy recovery via incineration and landfill.^34^ Research at UoN has explored biological recycling of polyethylene (PE), which involves the use of microorganisms or enzymes to degrade the plastic into valuable commodity chemicals.^35^ Biological recycling for PE is at a very early stage, with researchers still discovering microorganisms and the key enzymes.^36,37^ However, this technology has been successfully commercialized for polyethylene terephthalate (PET) plastic by Carbios in France. Carbios have demonstrated the degradation of plastic bottles and textile waste using their proprietary PET degrading enzymatic process and are currently constructing the first commercial plant.^38^

This new plastic recycling technology has potentially a huge sustainability impact, satisfying at least six of the UN SDGs. Less mismanagement of plastic waste would address *Clean Water and Sanitation* (SDG 6), *Life Below Water* (SDG 14) and *Life On Land* (SDG 15). Incorporating plastic into the circular economy would also reduce reliance on virgin petroleum-based plastic, addressing *Climate Action* (SDG 13), *Responsible Consumption and Production* (SDG 12) and *Sustainable Cities and Communities* (SDG 11).

Although the technology has advanced in recent years, transitioning to a bioeconomy and realizing the full sustainable potential of this technology will require many strategic partnerships. Biological recycling must overcome significant infrastructure challenges, such as the lack of physical infrastructure and efficient plastic waste collection and processing methods. However, process case studies taken from the food industry and biomass valorisation industry demonstrate the successful implementation of biobased production plants.^39,40^ Recent studies have also highlighted unfavorable investment conditions and market environment as economic transition barriers.^41^ These barriers involve a diverse range of stakeholders and discourage industrial collaboration to commercialize biological recycling of plastic.

### 2.4 Sustainable Production of Ammonia to Decarbonize the Energy System

Green ammonia has emerged as a promising candidate for carbon-free fuel, offering several advantages that position it as a potential cornerstone of future sustainable energy systems. Its high energy density, compatibility with existing green hydrogen technologies, and the presence of established global distribution networks make it an attractive option for decarbonizing various sectors, including transportation, industry, and agriculture.^36^ The realization of this technology could make significant contributions to multiple UN SDGs but directly supporting the drive toward *Affordable and Clean Energy* (SDG 7), by facilitating the transition to renewable energy systems.

However, the widespread implementation of green ammonia faces significant challenges. Chief among these is the need to dramatically increase production rates to meet potential future demand.^42^ This requirement necessitates substantial improvements in efficiency, a goal that has remained elusive for over a century due to the chemical and economic complexities involved in ammonia synthesis.^43^ Recent advancements in plasma technologies offer a promising new direction for green ammonia production.^44^ However, to fully realize the potential of these new technologies and drive innovation at the pace required to meet sustainability targets, fostering partnerships and collaboration across various sectors is crucial. The development and implementation of green ammonia technologies requires a multifaceted approach involving academia, industry, and government. Research institutions must work closely with industrial partners to ensure that laboratory-scale discoveries can be effectively scaled up to meet the demands of large-scale manufacturing plants. This transition from research to large-scale production is a critical challenge that requires careful navigation of technical, economic, and regulatory landscapes.

Government policies play a pivotal role in facilitating this transition. This may involve the development of funding initiatives, market incentives and regulatory frameworks to support the industry. One of the key challenges in implementing these policies is balancing the need for rapid innovation with the realities of industrial scale-up and economic feasibility. Local, regional, and national governments must work in concert to create a coherent policy framework that supports green ammonia development. The global nature of the ammonia market also presents challenges in terms of policy harmonization and international competition. Countries may need to balance their desire to lead in green ammonia technology with the need for international cooperation to create a viable global market.

By fostering innovative partnerships, supporting cutting-edge research, and implementing thoughtful and flexible policies, governments can play a crucial role in accelerating the development and adoption of green ammonia technologies. Success in this endeavor has the potential to significantly contribute to global sustainability targets and pave the way for a cleaner, more sustainable energy future.

## 3 Key Challenges and Solutions to Partnerships

The UoN hosted a workshop during the official preconference session at the Times Higher Education (THE) Global Sustainable Development Congress, in Bangkok, Thailand between 10th-13th June 2024. Workshop attendees were from both academic and nonacademic sectors; at all stages of their careers; and represented multiple geographies. Informal round table discussions with workshop attendees shaped and contributed to the following section of this perspective, which discusses in more detail many of the challenges related to conducting collaborative research.

Several themes surrounding partnerships evolved from these discussions, including different challenges alongside solutions to overcome such challenges. Alongside a summary of these topics, there are numerous best practice examples available which highlight the diversity of practical solutions to address the complex challenges, ranging from individual to multistakeholder efforts. The following section of this paper does not aim to encompass the full breadth of the discussions, or expose all challenges in collaborations for achieving SDGs, but rather hopes to cast light on some of the areas of interest.

### 3.1 Partnership Dynamics and Coordination

#### Challenges

Partnerships are key to achieving the SDGs, as they accelerate progress and implementation of sustainable technologies. However, one of the first barriers to overcome is the difficulty to successfully engage with collaborators. This can be for several reasons such as lack of access, diverse stakeholder interests or poor communication. There may be a reluctance from local partners to engage with certain projects and areas of research, especially those with a sustainability focus. Such perspectives and experiences maybe be more predominant within the Global South, where many countries are facing multiple overlapping crises and are struggling to prioritise sustainability.^45^ This issue of overlapping crises can lead to diverse stakeholder interests, where each party comes to a project from a different perspective. While these diverse perspectives can be beneficial, especially for multidisciplinary projects, and lead to a range of solutions, it can also lead to conflicting interests. These conflicting interests are often made more challenging if there is poor communication between the stakeholders. A silo mentality, where everyone works in isolation and focuses solely on their own goals, often sets in and can hinder the progress of an entire project.

Successful partnerships are cemented by trust and respect.^46^ Unfortunately, in scenarios in which trust and respect is lacking between collaborators, it can be challenging to generate equal benefits and opportunities for all members of the partnership. Furthermore, discussion topics arose related to partnership dynamics and coordination, particularly within sustainability, highlighting the imbalanced dynamic was especially prevalent within Global South and Global North collaborations. Such asymmetries in power within such research collaborations have been researched and reported by authors within the literature in different settings, including making conclusions regarding the importance of building trust.^47,48^ The Global North has exploited the Global South for its natural resources, resulting in climate change heavily affecting the Global South. This unequal exchange sees the majority of the economic benefits retained by the Global North, with the Global South bearing much of the environmental cost, perpetuating existing inequalities while also creating new ones.^49^ Relationships may be further damaged by misinterpretation of data for individual gain. For example, some workshop participants shared concerns that Global North and Global South partnerships were often used as a marketing tool to promote sustainability activities by the Global North partners, while Global South partners received unequal access to the benefits. In short, they felt that they were used as a form of greenwashing, leading to decreased desire to collaborate in the future.

#### Solutions

The use of media and online networks can overcome access barriers to collaboration, this use of technology facilitates easy implementation and avoids limiting access to only those privileged to travel to in person networking activities. Platforms like LinkedIn and other more formal networks can become invaluable tools, creating a nexus between related researchers and forging new links between disciplines and institutions. These networking opportunities facilitate collaboration, especially interdisciplinary connections, fostering more integrated thinking and enabling the discovery of experts who can fill gaps within partnerships. Networks centered around common goals, such as sustainability, make it easier to find partners with aligned purposes and advocacies. The presence of and frequency of both formal and informal networking opportunities also leads to greater trust between potential partners. Workshop participants felt that with regular communication before collaboration, trust could more easily be developed, leading to better partnership outcomes. Additionally, communication can be enhanced through more training and workshops focused on effective science and cross-cultural communication.

Mentoring programmes are one example of promoting professional development, and foster interdisciplinary collaborations, which can include improving communication skills. A summary of benefits and best practices for mentoring programmes has been published by the UK Research and Innovation, with the Royal Society also offering career development opportunities in these areas.^50^ Effective communication of the small wins in both a scientific manner and a simpler ley manner can go a long way in demonstrating feasibility, raising awareness and building momentum and trust within partnerships.

One strategy to avoid unequal collaborations between Global South and Global North actors and establish mutually beneficial partnerships may involve generating a list of best practices or a framework of global recommendations limiting the exploitation of researchers in the Global South. There are organisations, specifically global health organisations, actively involved in promoting ethical considerations in research collaborations.^47^ Recommendations could include shared authorship standards and guidelines, the promotion of “collaboration champions” that demonstrate effective and equitable collaboration practices, and frameworks and policies that ensure equal benefits for all partners. Although, the implementation and enforcement of these standards could be difficult, particularly in those countries with unstable governments. Therefore, this would rely on the individuals within the collaboration agreeing to these practices prior to collaborating and trusting that they will be upheld. This is particularly important for collaborations with many different stakeholders, agreements should be made at the early stages of projects around key decisions, such as dissemination of findings between partners.^51^

Trust can be reestablished by increasing effective stakeholder communication, particularly by acknowledging individual goals, perspectives and negotiating specific common objectives. This is especially important when the collaboration has a power imbalance. It was also suggested that there is a need for those in the more privileged position to strongly emphasize and demonstrate the equal opportunities/mutual benefits from the partnerships from the outset.

A final key component is to increase community engagement through greater dissemination of science and sustainability efforts. Some workshop participants felt that there is often a strong desire to collaborate, especially on issues around sustainability. However, these participants shared that access to recent science and research findings was often difficult, with paywalls and other barriers to entry preventing access. Improving the dissemination of research could potentially result in more collaboration opportunities and community engagement, particularly when collaborating with stakeholders across power imbalances and geographical locations.

#### Case Studies

At UoN there are several programmes and training opportunities which can be used to demonstrate how improved communication can lead to impactful sustainability partnerships. The University of Nottingham Interdisciplinary Centre for Analytical Science (UNICAS) awards provide funding for collaborative cross-disciplinary research and use of equipment and analytical expertise. Sandpit sessions are regularly organized to provide an informal networking opportunity for researchers from different faculties, schools and departments.^52^ Engineering and Physical Science Research Council (EPSRC) fund a sustainable chemistry center for doctoral training (CDT) at UoN. The CDT delivers cohort-based training which embeds principles of trusted research and environmental sustainability. The projects funded are often multidisciplinary and involve industrial partners, which facilitates a collaborative mindset for the CDT funded ECRs.

### 3.2 Resources and Funding

#### Challenges

Conducting rigorous research can be incredibly time intense, and hence time limitations are often cited as a barrier to establishing and maintaining partnerships. In different global contexts and for those at different career stages there are different time pressures on researchers, and this is especially prevalent for ECRs. The definition of ECRs varies across different geographies. For example, as stated in a report by the International Network for Advancing Science and Policy (INASP), the phases of an academic career are less defined in many low- and middle- income countries. In the Global South, many researchers have significant other teaching and administrative responsibilities, with PhDs often completed later during a researcher’s career.^53^ Whereas in many countries in the Global North, such as the UK, ECRs are typically defined as those in postdoctoral positions, generally less than 10-years post award of PhD.^54^ This difference needs to be considered when discussing time and resource availability, in terms of both challenges and solutions for establishing partnerships.

Time pressures on researchers are also closely linked to a lack of funding available for research only activities. This is highlighted by INASP as being a significant barrier to collaborations within research, with ∼90% of researchers stating lack of funding being a barrier to collaborations being at least of moderate consideration.^55^ Away from academic institutions, for example in partnerships involving nongovernmental organisations (NGOs), communication surrounding ongoing research can be time-consuming if not part of an employee’s main role responsibilities.

#### Solutions

Solutions for researchers to be able overcome resource limitations and allow the establishment of partnerships are multifaceted. For example, researchers time needs to be protected to focus exclusively on research to allow for adequate development of partnerships, especially for those with otherwise significant teaching and administration responsibilities. This limitation could be targeted through the availability of research grants for not only conducting research but also for dissemination of research. This latter activity of research dissemination, such as conference attendance and presentations, is vital for the communication of research excellence and establishing new collaborators for the continuation of research. Including research dissemination responsibilities within research grants would also go a long way in equalizing relationship imbalances as discussed in section 4.2.

However, the short-term length of some research grants and projects is also considered to be a barrier to effective partnerships. For example, projects coming to an end may result in relationships ending which can then not be maintained in subsequent research. This can also result in a higher turnover of researchers working on a project, which can negatively impact longevity of partnerships. The need for long-term funding is reported as being crucial for sustaining equitable partnerships.^56^

Finally, having dedicated networks specifically for ECRs, to allow for connections between researchers which does not include a significant time or travel requirement is also a solution to allowing establishment of partnerships. Such networks are available, including those which are specific chemistry researchers, such as two recently established networks of the Institute of Chemistry of Ireland Young Chemists Network (ICI-YCN) and the Thailand Younger Chemists Network (TYCN).^8^

#### Case Studies

Some institutions have found solutions to being able to provide protected research time. For example, a policy, known as “Deloading” takes place in all higher educational institutions within the Philippines, which allows staff members to reduce their hours of teaching allocation in order to spend more time conducting research, among other activities. However, this also needs to be well managed and supported to be successful, as it has been highlighted that only 18% of researchers had time “Deloaded” in a 2016 report.^57^

A further example of solutions in action is the establishment of the The Young Aging Crew (YAC). The Young Aging Crew is a collection of ECRs from five continents who come together regularly (both virtually and in person at conferences) to discuss research in aging phenomena of asphalt pavements.^58^ One of their initial goals is to collaborate and introduce other members to each other’s research. It may be possible for such initiatives to be setup for other areas of research to allow ECRs to gain confidence in collaborations without significant time or travel requirements.

### 3.3 Cultural and Social Barriers

#### Challenges

Partnerships addressing the SDGs face numerous cultural and social barriers that can hinder their effectiveness. A major challenge discussed was limited access to travel due to restrictions, visa issues, and conflicts or security concerns, which limit collaboration opportunities for researchers in certain regions. Research has highlighted this to be an issue disproportionately impacting researchers from low-income and middle-income countries.^59^ In addition to this, such issues have been picked up in mainstream media in recent years.^60−62^

Language barriers may also be a major challenge to collaborative partnerships. For example, the wide array of primary languages spoken in Africa has been reported as a barrier to open science and formation of collaborative networks across the continent.^63^ While language barriers are particularly problematic in multilingual countries and regions, they also exacerbate already difficult partnerships when the primary language of collaboration is not their first/native language.

Power imbalances between partnering institutions were emphasized as an additional complicating factor for collaborative relationships, with concerns especially arising from institutions in the Global South about being exploited by partnerships with the Global North. In addition to power imbalances between institutions, there can be a lack of respect for certain academic disciplines even within an institution. These can lead to systemic superiority complexes that stifle the acceptance of new interdisciplinary ideas. Differing cultural standards between countries were also discussed, such as how negative news is communicated, expectations for proactive engagement in collaborative relationships, or simply preferred methods of contact. A lack of understanding of these cultural aspects can significantly hinder how effective collaborations can become.

#### Solutions

Overcoming these varied barriers requires targeted solutions. Cultural competence training is one method to equip individuals with the skills needed to navigate diverse cultural landscapes effectively. Integrating linguistic and cultural orientation sessions can help bridge communication gaps and foster mutual understanding among partners. These orientation classes can support not only effective communication between countries, but also between those from the same county with different socioeconomic/cultural/language backgrounds. A potential limitation would be the resources available for running these sessions.

The empowering of local leadership and engaging community leaders was emphasized to ensure that collaborative sustainability research initiatives are relevant and embraced by the communities they aim to serve. Indigenous group partnerships and the inclusion of sustainability education with indigenous groups can promote sustainable practices that are culturally appropriate and impactful. Involving the community from the outset was discussed to ensure that science and engineering initiatives are tailored to local needs and that the community is invested in the outcome but does require the local community to be interested in engaging. Illustrating the tangible value that sustainability can bring, including profitability, can help garner wider support. NGOs and similar agencies were highlighted to play a pivotal role in bridging gaps between communities, academia, government, and industry, facilitating smoother collaborations. By adopting these suggested solutions, embedding sustainability into core ideas and demonstrating its benefits, partnerships can overcome cultural and social barriers and effectively contribute to the achievement of the SDGs.

#### Case Study: Live-in-Laboratories

The ‘Live-in-Laboratories’ program at Amrita university in India is an exemplary case study for overcoming cultural and social barriers in partnerships aimed at addressing the UN SDGs.^64^ Here we highlight a few aspects of the program which may be useful to others seeking to address similar challenges:

- **Interdisciplinary projects**: projects under the ’Live-in-Laboratories’ program are interdisciplinary, involving fields like engineering, healthcare, social sciences, and environmental studies. This broadens the scope of impact and brings together varied expertise to address complex issues related to the SDGs.
- **Immersive learning and collaboration**: the program involves students and faculty from diverse backgrounds living and working in rural Indian villages. This immersive experience fosters mutual understanding and respect, bridging cultural and social divides.
- **Community-centered approach**: by focusing on the needs and priorities of local communities, the program ensures that solutions are culturally appropriate and socially acceptable. This approach helps in gaining the trust and cooperation of local populations.
- **Capacity building**: the program emphasizes skill development for both students and local residents. This two-way learning process empowers communities while sensitizing participants to the cultural and social contexts they work within.
- **Sustainable solutions**: projects are designed with sustainability in mind, ensuring long-term benefits for the communities. By involving locals in the implementation and maintenance of solutions, the program promotes ownership and continuity.
- **Cross-cultural exchange**: participants from different countries and regions engage with Indian rural communities, leading to a rich exchange of ideas and practices. This cross-cultural dialogue helps dismantle prejudices and fosters global citizenship.

Overall, the ‘Live-in-Laboratories’ program exemplifies how immersive, community-engaged projects can effectively overcome cultural and social barriers, fostering partnerships that advance the UN SDGs in a locally relevant and sustainable manner.

### 3.4 Governance and Policy

#### Challenges

Perhaps the most unifying theme from these workshop discussions was the role of governance, policy and large-scale partnerships in achieving the SDGs. One of the primary challenges in governance is aligning multiple, often conflicting goals. Policymakers must balance environmental sustainability with economic growth, energy security, and social equity. For instance, rapid deployment of new green technologies might accelerate decarbonization but could also lead to job losses in traditional industries or increased costs for consumers. Governments must navigate these trade-offs carefully, often facing pressure from various stakeholders with divergent interests.

While the importance of government policies toward the SDGs was overstated, the nuance of creating balanced policies that encourage innovation while ensuring safety and environmental protection was a significant concern. Overly rigid regulations can hinder technological progress and discourage investment in novel green solutions. Conversely, lax oversight could lead to safety hazards or long-term environmental damage. Policymakers must strike a delicate balance, implementing adaptive regulatory frameworks that evolve with technological advancements while maintaining necessary safeguards.

The public’s growing scepticism toward “greenwashing,″ presents an extra hurdle and can extend to government initiatives promoting green technologies. To overcome this, policymakers and industry leaders must prioritize transparency, provide clear and verifiable information about the benefits and limitations of new technologies, and demonstrate tangible progress toward sustainability goals.

International cooperation, a key aspect of SDG 17, adds another layer of complexity. Different countries may have varying priorities and capabilities in developing and implementing green technologies. Harmonizing standards, sharing knowledge, and equitably distributing the benefits and burdens of this transition require careful diplomatic navigation and robust international frameworks.

#### Solutions

To address the challenges associated with implementing green and innovative technologies, several potential solutions can be explored, focusing on public engagement, government incentives, and early governmental involvement.

Government incentives can play a significant role in promoting green technologies many industries that are in most need of innovation often have the highest barriers toward implementing change and often due to the lack of profitability in making strides toward SDGs. In this case government incentives provide the necessary push toward sustainability counteracting the inertia of historic institutions and “tried and tested” ways of working. These could include tax breaks for companies investing in green technologies, subsidies for consumers using eco-friendly products, or grants for research and development in sustainable solutions.

Early governmental involvement was also highlighted as crucial in shaping public perception and policy around new technologies bringing them to the forefront of policy discussions. Appointments of government ministers in key strategic areas could facilitate rapid deployment of innovation by enabling government bodies to be well-informed about the latest progress, advocate for supportive policies, advise on associated governmental barriers at the earliest stage and serve as bridges between the scientific community, industry, and policymakers.

Government initiatives to promote green technologies can be multifaceted and far-reaching. Establishing a network of partnerships within government structures extended toward academic institutions ensures sustained advocacy and informed policy decisions. Creating platforms for ongoing dialogue between scientists, policymakers, industry leaders, and the public fosters collaboration and knowledge exchange. Finally, integrating green technology education into school curricula cultivates long-term public engagement and understanding, laying the groundwork for a more environmentally conscious society.

#### Case Studies

##### Public Engagement

Improving the dissemination of scientific information to the public is crucial for building trust and engagement. One effective approach is the 3 min thesis (3MT) competition model, originally developed by the University of Queensland, Australia for PhD students to present their research concisely to a nonspecialist audience.^65^ This concept has been replicated globally due to its success in bridging the gap between state-of-the-art research and a layman audience. This concept can be adapted for green technologies, where experts and innovators present their ideas in brief, accessible formats. Such events could be organized at local, national, and international levels, potentially televised or shared online, to reach a broader audience. This approach helps demystify complex technologies, making them more relatable and understandable to the general public.

##### Government Incentives

India’s Waste to Wealth campaign, launched in 2014, serves as an excellent example of how government-led initiatives can drive change.^66^ This comprehensive program aimed to improve sanitation and waste management across the country by combining a robust policy framework with technological advancements. The campaign utilized innovations such as automatic waste segregators and implemented circular initiatives, using segregated waste as feedstock for gasifier and pyrolysis units to generate energy. This multipronged approach demonstrates how policy, technology, and public engagement can be effectively combined to address complex environmental challenges.

By implementing these solutions, governments can foster a more informed and engaged public, create a supportive environment for green innovations, and ensure that policy decisions are grounded in scientific understanding and long-term sustainability goals. Therefore, contributing to multiple SDGs while navigating the complex landscape of sustainable development.

## 4 Future Outlook

It is clear that SDG 17 and the activities surrounding it are vital to allow the diverse goals which underpin the success of all SDGs to be reached. Within sustainable chemistry and engineering it is well recognized that strong collaborations between different academic disciplines, industry partners and policy makers are required to reach positive outcomes in many areas such as atmospheric CO_2_ removal, clean energy or pollution reduction. This can also be understood by the framing of sustainability itself as a partnership between three pillars, economic, social and environmental factors.

Moreover, examples of successful collaborations and partnerships can be found in far spanning and often considered unrelated areas of research. Interpersonal relationships are central to successful collaborations, and as these span far beyond academic disciplines and the sciences, so too do the barriers and associated solutions. This is particularly important for researchers in the early stages of their careers, who are pivotal to both conducting current research activities and to shaping the research landscape of the future.

This paper highlights the importance of bringing together diverse groups of stakeholders not only to work on cutting edge research together, but also to discuss how research can be conducted. Examples of best practice have been presented for individual researchers to consider and distill in the lens of their own research setting. Broadly practices need to be developed which result in open, trustworthy and fair partnerships. To achieve this, changes can be made on different scales, from individual initiatives, such as virtual interest groups, through to large national level policy decisions, such as early governmental involvement and incentives.

As a final call to action, we encourage others to facilitate similar multidisciplinary working groups to continue to share solutions and best practices in collaborating for the SDGs. Such events need not be large, costly, in-person events, but advantage should be taken of the availability of digital tools designed for an ever more virtual and connected world. Such actions will fundamentally contribute to chemistry and engineering’s vital input toward reaching the agenda of the UN Sustainable Development Goals.
